# Heart Failure in East Asia

**DOI:** 10.2174/1573403X11309020004

**Published:** 2013-05

**Authors:** Yutao Guo, Gregory YH Lip, Amitava Banerjee

**Affiliations:** 1 University of Birmingham Centre for Cardiovascular Sciences, City Hospital, Birmingham, United Kingdom; 2 Department of Geriatric Cardiology, Chinese PLA General Hospital, Beijing, China

**Keywords:** Heart failure, epidemiology, management, East Asia.ords

## Abstract

Heart failure (HF) carries a major burden of disease in East Asia, with high associated risk of mortality and morbidity. In recent decades, the epidemiology of HF has changed with social and economical development in East Asia. The burden of HF is still severe in East Asia.

The prevalence of HF ranges from 1.3% to 6.7% throughout the region. As aetiological factors, ischaemic heart disease has increased and valvular disease reduced in most East Asian countries. Diuretics are the most commonly used drugs (51.0%-97%), followed by renin-angiotensin system (RAS) inhibitors (59%-77%), with angiotensin-converting enzyme inhibitors, ACEI, (32%-52%) and has angiotensin-2 receptor blockers, ARBs (31%-44%) in similar proportions. β-blocker use has also increased in recent years.

Total mortality from HF ranges from 2% to 9% in China, Taiwan, Singapore, Thailand, and Japan. Age>65 years, diabetes mellitus, anaemia, renal dysfunction and atrial fibrillation (AF) are associated with adverse outcome. More prospective, region-specific data are still required, particularly regarding new drug therapies such as eplerenone and ivabradine.

## INTRODUCTION

Heart failure (HF) is increasingly prevalent worldwide and is associated with significant morbidity and mortality. The lifetime risk of developing HF is one in five at the age of 40 years [1]. Over 5 million individuals in the United States have HF, and more than 550, 000 are diagnosed annually [2]. It is becoming a burden in health care, which results in greater $39 billion annual cost in North American and £716 million (1.8% of the total NHS budget) in the United Kingdom [3]. HF is a global epidemic, but clinical characteristics and treatment may vary for patients with HF across geographic regions [4]. 

In China, 4.2 million individuals have HF, and cardiovascular disease is the leading cause of death [5-6]. In Japan, 1.0 million individuals were estimated to have HF in 2005, and this figure is expected to rise to 1.3 million by 2035 [7].

During the last 20 years, changes in society and economy in East Asia countries, such as the aging of the population and medical progress, have led to changes in the epidemiology of HF [8]. Some region-specific, epidemiological studies have been conducted in East Asia, especially in China and Japan [9-26]. These research studies included the populations in both urban and rural areas, and investigated the epidemiology of different age-groups of patients. 

The present review based on recent evidence, aims to update the epidemiology and management of HF in East Asian countries, including the prevalence/incidence, aetiology/comorbidities, treatment, prognosis, HF with preserved ejection fraction (HFPEF), and HF in older patients in East Asia.

## SEARCH STRATEGY

We searched PUBMED between 1 January 1990 and 30 June 2012 for articles concerned with the epidemiology of HF, risk factors for HF, drug treatment and prognosis of HF in populations in East Asia. The search terms were: HF, PEF, prevalence, incidence, aetiology, risk factor, drug management, prognosis, outcome, mortality, rehospitalization, China, Chinese, Japan, Japanese, Taiwan, Hong Kong, Korea, Mongolia, Malaysia, Singapore, Thailand, East Asia or the combination of these key words. We included epidemiological studies and clinical trials. Study populations of fewer than 720 people and duplicates were excluded. The articles meeting the eligibility criteria with full text were selected for data extraction. A total of 324 articles were retrieved by literature searches for reports on the epidemiology and treatment of HF in East Asia. Of these, 42 articles qualified for inclusion.

## PREVALENCE AND INCIDENCE OF HF

The prevalence of HF was 6.7% in Malaysia, 4.5% in Singapore, and 1.3% in China respectively [16, 19, 20]. The reported incidence of HF in the Chinese population is 0.7 to 0.9 per 1000 [13, 15]. There are 1.8 million individuals with congenital cardiac abnormalities in China and 500, 000 new cases of HF every year [27]. A hospital-based study showed that the prevalence of Chinese patients with HF by age < 40, 40 – 49, 50 - 59, 60 – 69, and 70 - 79 years was 6.7%, 10.7%, 18.8%, 23.5%, and 30.8% respectively (p<0.01), while community-based studies demonstrated approximately doubling prevalence with each decade of age with 0.3% at age 35- 44 years, 0.6% at age 45-54 years, 1.3% at age 55-64 years, 2.6% at age 65-74 years, and 4.1% at age ≥75 years [11, 13]. On the other hand, the age of Chinese patients admitted for HF has significant reduced in recent years: 66.4 ± 14.1 years between 2000 and 2003, 64.9 ± 14.4 years between 2004 and 2006, and 64.2 ± 14.8 years between 2007 and 2010 (P < 0.01) [11]. Age-adjusted hospital admission rates for HF increased by 38% in 1990s in Singapore [19], and hospital admissions for HF were about 35% higher in Malaysians than in Chinese in this study [19]. 

## AETIOLOGY AND COMORBIDITIES OF HF

In recent decades, the ischemic aetiology for HF has increased in East Asia populations but not as greatly as in Western populations. In a USA national registry, out-patients with HF had a relatively higher proportion of ischaemic aetiology (52.6%, 71.1% and 72.9% for those aged <65, 65-76 and >76 years, respectively) and prior myocardial infarction (MI) (34.3%, 42.8% and 41.5% for the respective ages) [28]. Asian Americans were reported to have higher prevalence of HF after acute MI [29].

**Fig. (1):**
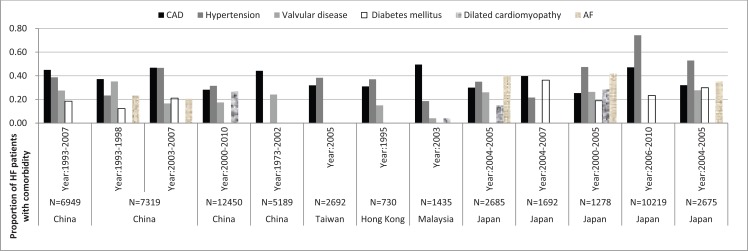
Aetiology and comorbidities in studies of HF in East Asia. CAD: coronary artery disease. AF: atrial fibrillation.

Aetiology and comorbidities of HF in East Asia are given in Figure 1. The proportion of coronary artery disease (CAD) ranged from 25% to 47% in HF populations in mainland China, Taiwan, Hong Kong, Malaysia, and Japan [9-26]. Hypertension was the most likely comorbidity in Japanese patients with HF, with the highest rate of 74% [25], however, the lowest rate of hypertension (19%) was in Malaysia [15]. The prevalence of hypertension in HF was 23%-47% in mainland China [9-11, 14], 38.3%-41.3% in Taiwan [15], and 37% in Hong Kong [10]. Moreover, valvular disease appears to be a less common aetiology of HF in East Asia, reported in 15% - 35% of patients with HF in most of countries, but only 4% in Malaysia [18]. Diabetes and atrial fibrillation (AF) are also common comorbidities, with 12%-36% and 21%-42% prevalence respectively among HF patients [9-26]. Furthermore, renal dysfunction and anaemia are risk factors of HF. A hospital-based study of patients with HF in Canada indicated that Chinese patients had the highest rates of renal disease compared to white populations [30]. 

Data about comorbidities of HF were consistent in one community-based study and three hospital-based studies in China [9-11, 14]. The most common comorbidities were CAD, hypertension, and diabetes mellitus. Dilated cardiomyopathy, valvular heart disease, and AF were common comorbidities in these Chinese populations with HF. A 10-year survey of hospital in-patients with HF showed rheumatic valvular heart disease was the most frequent cause in patients aged less than 40 years old and dilated cardiomyopathy was most likely in patients aged 40-59 years [11]. Renal dysfunction was also common in Chinese patients with CHF, and the China Heart Survey showed that 34.3% patients with CHF had chronic kidney disease [12]. AF was another main cause of Chinese HF patients in 1990s and early 2000s, with the rate of 20.6%-23.2%9. 

Two Japanese studies (CHART-1, CHART-2) showed that an increasing trend towards ischemic aetiology (26.4%-47.1%) and comorbidities with diabetes (19.5-23.3%) and hypertension (47.4%-74.3%) in Japanese HF patients [20, 24, 25, 31], confirmed by the JCARE-CARD which revealed a prevalence of CAD in 32% of patients [22]. In the CHART-1 study between 2000 and 2005, CAD accounted for only 26.4% and the proportion of patients older than 65 years was 66% [20], but in the CHART-2 study between 2006 and 2010, CAD as a comorbidity increased to 47.1%, hypertension was 74.3%, and diabetes was 23.3% [22]. Additionally, JCARE-CARD showed that 57% patients with HF had anaemia [23], and the prevalence of AF was common (40%) in hospital-HF patients [21]. 

## DRUG TREATMENT FOR HF IN EAST ASIA 

**Fig. (2):**
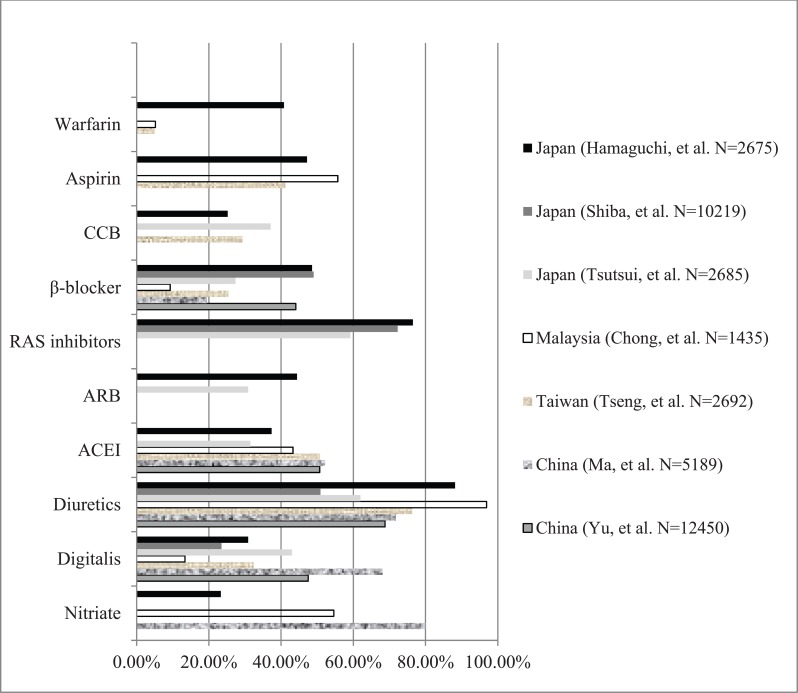
Drug treatment or HF in East Asia. CCB: calcium channel blocker; ACEI: angiotensin-converting enzyme inhibitor; ARB: angiotensin- converting enzyme receptor inhibitor; RAS: renin-angiotensin system.

In East Asian countries, diuretics were the most common treatment, used in between 50.9% and 96.9% of patients, followed by renin-angiotensin system (RAS) inhibitors (59.2-76.5%). Prevalence of angiotensin-converting enzyme inhibitor (ACEI) and angiotensin-2 receptor blocker (ARB) use was 31.5-52.2% and 30.9-44.4% respectively. Nitrate use was variable (23.3-80.0%), and β-blocker use varied from 19.5-49.0% in China, Taiwan, and Japan, to only 9.3% in Malaysia. Aspirin was used in 41.2% of HF patients in Taiwan, 47.2% in Japan, and 55.7% in Malaysia. Warfarin was reported to be used in 40.8% Japanese patients with HF [9-26] (Fig. **2**). The relatively high rates of aspirin and warfarin usage may reflect the underlying high prevalence of CAD and AF in these populations, but population-based data are lacking. 

Nitrates, diuretics, digitalis, ACEI, and β-blocker were the most commonly used agents in Chinese patients with chronic HF. Nearly half of patients with NYHA (New York Heart Association) Class III-IV were treated with the combination of ACEI and β-blocker, or diuretic, digitalis, ACEI and β-blocker. β-blocker therapy was most likely used in patients with NYHA Class II-III [14]. Although the administration of ACEI and β-blockers increased in recent years, their application in clinical practice did not achieve the level proposed by recent guidelines for HF management [32, 33]. Diuretics were the most commonly used drugs, in both younger (74·4%) and older (76·9%) patients in Taiwan, followed by ACEI and/or ARB and aspirin15.

β-blocker use increased in the last decade, whilst diuretics and digitalis decreased in Japanese cohorts with HF. The usage of RAS inhibitors and β-blockers for overt HF patients in the CHART-1 and CHART-2 studies increased from 69.1% to 72.3% and from 27.9% to 49.0%, respectively. In contrast, the usage rate of loop diuretics and digitalis decreased from 76.3% to 50.9% and from 48.1% to 23.5%, respectively.

New pharmacological therapies proposed by recent consensus guidelines for the management of HF [33], such as eplerenone and ivabradine, are less likely to be used in clinical practice in East Asia. There is very limited research data on these drugs in East Asian patients, who are under-represented in recent clinical trials in HF, e.g. only 8% of Asian patients in the SHIFT trial [34-35].

## ANTITHROMBOTIC TREATMENT IN HF IN EAST ASIA

The comorbidities related to HF (such as CAD, AF) lead to a high risk of thromboembolic events, including stroke, pulmonary embolism and peripheral arterial embolism, whilst coronary ischaemic events also contribute to the progression of heart failure. CAD as a major aetiology of HF has increased with the societal and epidemiologic transition in East Asian countries and the prevalence of AF among patients with HF was 21-42% in those countries [9-26]. In a Japanese cohort study, 15% of HF patients were reported to suffer stroke [23]. The rate of aspirin use was consistent across Japan, Malaysia, and Taiwan (41.2-55.7%) [15, 18, 23]. 

Warfarin was used in 40.8% patients with HF in Japan, while 35% patients had AF and 15% patients suffered the stroke in this cohort [23]. However, only 5.1-5.2% patients with HF used warfarin in Malaysia and Taiwan [15, 18]. There were limited epidemiologic data from East Asia to evaluate the prevalence of comorbidities related to HF which may increase risk of thrombosis. Further research is required to provide such data in order to inform use of antithrombotic treatment for stroke prevention in HF patients.

To date, four major randomized controlled trials of antithrombotic therapy in HF have been performed [36-39]. There was no significant overall difference in the composite end point of ischemic stroke, intracerebral hemorrhage, or death between warfarin and aspirin in WARCEF and there is no evidence to support routine anticoagulant therapy in HF patients without AF. As in other clinical trials, Asian patients are greatly under-represented.

## DEVICE THERAPY

Cardiac resynchronization therapy (CRT) and implantable cardioverter-defibrillator (ICD) therapies have not been widely used in treatment of HF in East Asia. CRT with defibrillator (CRT-D) became available in Japan in 2006 [40]. Large-scale epidemiological data regarding CRT-D, CRT-P and ICD are lacking in East Asian countries, and prospective data are required.

## CARDIAC REHABILITATION AND TELEMONITORING IN HF

There are scarce data on cardiac rehabilitation and telemonitoring or telemedicine in the management of HF in East Asia. A nationwide survey on the implementation of cardiac rehabilitation following acute myocardial infarction (AMI) showed that 0-9% of Japanese hospitals provided outpatient cardiac rehabilitation programs [41]. A Japanese study including 442 patients with AMI or coronary artery bypass graft surgery (CABG) suggested less favourable physiological and psychosocial outcomes in women than men [42]. Small studies suggest that short educational programmes in the outpatient setting can improve long-term outcomes in HF [43-44], but other studies show mixed results, and that cardiac rehabilitation may increase patient anxiety [45-47]. There may be a role for Chinese qigong exercise in cardiac rehabilitation [48].

## PROGNOSIS AND OUTCOME OF HF

Total mortality from HF was 2.2-8.8% in China, Taiwan, Singapore, Thailand, and Japan [19, 22, 23, 49]. 30-day mortality was similar in mainland China and Taiwan at 5.3% and 3.9% respectively [15]. In a 15-year trend analysis (from 1993 to 2007) in China, mortality of young patients with HF reduced [9], however, 30-day mortality in those individuals aged over 80 years increased from 9.8% to 20.0%. In this Chinese population, the 30-day hospital mortality showed a downward trend in HF patients with CAD, decreasing significantly from 9.3% in 1993-1997 to 3.8% in 2003-2007 (P<0.001) [50]. In another Chinese cohort with chronic HF, including 6453 patients with mean follow-up of 3 years, HF mortality was 30.1% in CAD, 44.9% in dilated cardiomyopathy (DCM), 36.2% in hypertensive heart disease (HHD), and 13.1% in rheumatic heart disease (RHD). Chronic systolic HF due to CAD, DCM and HHD carried a worse prognosis than that of RHD [51]. A higher hospital mortality rate was associated with an increased number of comorbidities10. Mortality from HF decreased from 7.3 per 10 000 in 1991 to 6.1 per 10 000 in 1998 in Singapore, and the decline was greater in women19. But, Indians and Malays had a worse outcome compared to the Chinese, probably due to higher mortality and HF readmission rates. A possible explanation for the worse outcomes in Indians could be due to the higher prevalence of diabetes mellitus and atherosclerotic vascular disease [52]. 

The rehospitalisation rate was 23.4% to 40% in three Japanese HF cohorts. In Thailand, the rehospitalisation rate within 30 days after discharge was 14.1% [50]. The length of hospital stay (LOS) > 5 days was the predictor of early readmission. Prognosis of HF in East Asia is shown in Table **1**.

The prognosis and risk factors of HF in East Asia may be comparable with Western populations [53], although prospective data are lacking. Although survival after HF diagnosis has improved over time in North America, the death rate remains high, with nearly 50% of people diagnosed with HF dying within 5 years1. The reported 30-day in-hospital mortality of patients with HF ranged from 1.7-7.2% in North America [54]. The 30-day, 1-year, and 5-year case fatality rates after hospitalization for HF were 10.4%, 22%, and 42.3%, respectively [55]. Three-fourths of all HF hospitalizations are due to exacerbation of symptoms in patients with known HF. One-half of hospitalized HF patients experience readmission within 6 months [56, 57].

Consistent with the data from North America, diabetes was a significant prognostic risk factor in Japanese population. Age was also associated with higher risks of adverse outcomes. In one study, 70% patients were over 65 years, and women were mostly over 70 years [21]. Anaemia and lower haemoglobin were independently associated with long-term adverse outcomes, including all-cause death, cardiac death, and rehospitalisation [22]. Japanese HF patients with reduced eGFR had a higher incidence of all-cause death and re-admission because of worsening CHF [24, 26], whilst renal dysfunction was independently associated with advanced NYHA classes in a Chinese population [12]. Ventricular tachycardia or atrial fibrillation could impact the prognosis of patients with HF [58].

Advanced HF has an extremely high mortality and morbidity and is associated with poor quality of life. Future research is needed to identify high-risk individuals in order to improve patient outcomes. 

## HFPEF IN EAST ASIA

The majority of data from East Asia regarding HFPEF is from Japanese studies. Several multicentre-studies demonstrated the prevalence of HFPEF from 26% to 45% in Japan [24, 59]. Patients with PEF (EF ≥50%) were more likely to be older and female. Hypertension and hypertrophic cardiomyopathy were more common in HFPEF, whilst MI or CAD were less common (JCARE-CARD data) [20-22]. Patients with HFPEF had higher prevalence of AF, and anaemia was also common (20-30%) and associated with higher mortality in HF patients. Renal failure and diabetes mellitus were the common comorbidities in patients with HFPEF. Calcium channel blockers (CCB) were used commonly in patients with HFPEF, whilst ACEI and β-blocker were less likely to be administered in those patients [60]. 

In-hospital mortality of HFPEF patients was 4.6% in Thailand and 2.2% in Singapore [50, 50]. CHART studies showed that all-cause mortality of Japanese patients with HFPEF at 1, 2, and 3 years was 7%, 16%, and 22%, respectively [24]. HFPEF patients had in-hospital mortality of 6.5%, which was not different from patients with reduced EF (<40%) after multivariable adjustment (JCARE-CARD data) [22]. There was no significant difference in survival analysis for all-cause mortality or cardiac mortality between patients with reduced and preserved EF. Rehospitalization rates were 23.7% and 25.7% in reduced and preserved EF patients respectively, with no difference between groups (p=0.47). 

## ELDERLY HF IN EAST ASIA

**Fig. (3):**
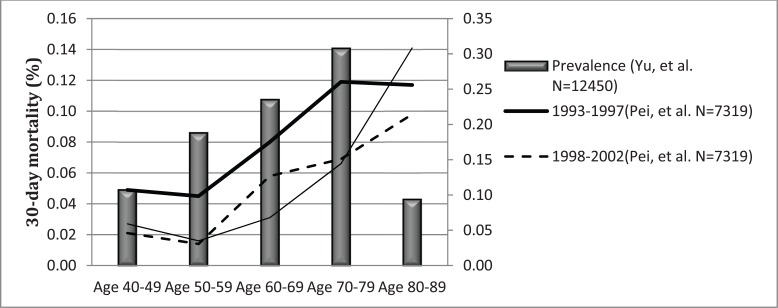
Prevalence and mortality of HF in Chinese population with age.

The prevalence of HF increased in proportion with aging. Hospital-based studies showed that the prevalence of HF in patients aged 60 years was 23.5-30.8% in China. The annual incidence of HF was 14 per 1000 in men and 20 per 1000 in women older than 85 years in Hong Kong [16], and 22 per 1000 population in elderly people in Taiwan [15]. Hypertension was the most common cause of patients aged 60 years in a Chinese HF cohort, and multiple comorbidities were predominant in the elderly compared to the younger patients [8]. Therapy with nitrates, ACEI and ARB was most common in patients aged over 60 years compared to the patients aged less than 60 years. Combination therapy with diuretic, digitalis, ACEI, and β-blockers was common in the elderly with CHF. β- blocker use was used with equal frequency in elderly and young Chinese patients with HF. Patients greater than 85 years have a 25-fold higher risk of heart failure hospitalization, longer length of in-hospital stay, higher total medical expenditure and higher in-hospital mortality [15]. The 1-year mortality rate increased with age, reaching 40% for patients older than 85 years in Hong Kong [16]. The mortality of young patients with HF reduced during recent years in mainland China, but the mortality of very elderly patients (aged over 80 years) did not significantly decrease (Fig. **3**).

The proportion of elderly population is high in Japan. The prevalence of HFPEF patients older than 65 years was 66% (CHART-1 study) [24]. 70% of patients with HF were over 65 years, and women with HF were mostly over 70 years in Japan [21]. Among patients aged over 70 years with HFPEF, valvular heart disease was the most frequent aetiology in CHART-1 study in the early 2000s. However, subsequent Japanese studies, including CHART-2, JCARE-GENERAL and JCARE-CARD, showed that hypertension in patients with HF increased in prevalence and became the most common comorbidity. Older age was also associated with the adverse outcome and 1-year mortality rate was 9.1% in HF patients with mean age of 74 years [21]. 

Further detailed data from individual studies regarding the epidemiology and management of HF in East Asia are shown in the Appendix.

## CONCLUSION

The prevalence of HF ranged from 1.3 to 6.7% in East Asian countries, with increasing prevalence with age. In terms of aetiology of HF, ischemic heart disease has increased and valvular disease decreased in this region during the last 20 years. Patients with HF and preserved EF were more likely to be older, female, and to have hypertensive heart disease. The mortality of HF remains high, and increasing age, diabetes, anaemia, renal dysfunction, and AF are associated with adverse outcome. Although the usage of ACEI, ARB, and β-blocker therapy has increased in recent years, diuretics, nitrates, and digitalis are the most commonly used drugs. The increased usage of ACEI, ARB, and β-blocker may reflect the increasing role of coronary artery disease as the aetiology of HF and increasing adherence to international guidelines. However, more evidence is needed in East Asian settings for novel drugs such as eplerenone and ivabradine. Antithrombotic treatment for prevention of thromboembolism in patients with HF in East Asia requires further research. 

## Figures and Tables

**Table 1.** Epidemiology and management of HF in East Asia

Study	Country and Setting	Population (n) Age, Mean (SD) Male, %	Study Design and Date	Prevalence/Incidence,% Mortality,% Rehospitalizaiton,%	Aetiology/comorbidities, %	Drug
Pei *et al*. 2011 [6]	China	7,319 CHF patients Age:59.6(16.9) Male:62.1%	Retrospective 1993-2007	30-day mortalities: 5.3% Male: 5.4% Female: 5.2%	1993-1998: Coronary heart disease: 37.2% Hypertension: 23.3% Diabetes mellitus: 12.3% Rheumatic vulvar disease:35.2% 2003-2007: Coronary heart disease : 46.8% Hypertension: 46.7% Diabetes mellitus: 21.1% Rheumatic vulvar disease: 16.6%	
Yin *et al*. 2011[7]	China Beijing (Urban)	6,949 patients with HF Male:4344 Female: 2605	Retrospective 1993-2007		Coronary artery disease: 45.0% Hypertension: 38.7% Valvular disease:27.5% Rheumatic valvular disease:21.8% Nonrheumatic valvular disease: 5.7% Diabetes mellitus: 18.3%	
Yu *et al*. 2011[8]	China Hubei (Urban, rural)	12,450 patients Age: 62 (15) Male: 7166, 57.6%	Retrospective 2000-2010 12 hospitals	<40 years: 6.67% (830/12 450) 40-49 years: 10.7% (1336/12 450) 50-59 years: 18.8% (2342/12 450) 60-69 years: 23.5% (2920/12 450) 70-79 years: 30.80% (3835/12 450) ≥80 years: 9.53% (1187/12 450) NYHA class I: 0.60%,II: 23.20%,III:50.31%,IV: 26.50%	Hypertension: 31.5% Coronary heart disease:28.2% Dilated cardiomyopathy: 26.6% Rheumatic valvular heart disease: 17.5% Aetiology: Age<40: rheumatic valvular heart disease Age40-59 : dilated cardiomyopathy Age≥60 years: hypertension	Digitalis :47.5% Diuretics:68.8% ACEI:50.7% β-blocker:44.1% aldosterone antagonist :53.1%
Liu, *et al*. 2011 [9]	China	1006 patients with CHF Age: 71.3(9.9) Male: 63.7%	Retrospective 2005		Chronic kidney disease: 34.3%	
Yang, *et al*. 2010 [10]	China Xinjiang (6 localities, random sampling)	8459 adults Age: 35 years over	Retrospective Communities-based	HF: 1.26% Male: 1.61%; Female: 0.93% (p<0.05) Age35- 44:0.29% Age45-54:0.60% Age55-64:1.32% Age65-74:2.55% Age≥75 years:4.10%		
Ma, *et al*. 2007 [11]	China	5189 patients with CHF Age: 62.9(13.4) Male: 49.5%	Retrospective 1973-2002		Aetiology Coronary heart disease:44.2% Rheumatic heart disease: 24.1% Pulmonary heart disease :19.0% Cardiomyopathy:4.8%	Nitriate:80.0% Diuretics:71.8% Digitalis:68.1% ACEI:52.2% β-blockers:19.5%
Tseng, *et al*. 2011 [12]	Taiwan	2692 patients with HF Age: 73(13) Male: 55%	Retrospective Random sample of insurant 2005	HF incidence: Age 20-60 years:88/100,000 Age≥65 years:21.8/100,000 In-hospital mortality: Total:3.9% Age 20-60 years: 2.7% Age≥65 years:4.2%	Comorbidities: Age≥65 years: Hypertension:38.3% Infection:32.0% Ischemic heart disease:31.9% Age 20-60 years: Hypertension:41.3% Diabetes:35.5% Ischemic heart disease:29.8%	Diuretics:76.3% ACEI:50.8% Aspirin:41.2% Digoxin:32.4% CCB:29.3% β-blockers:25.4% Clopidogrel:13.2% Warfarin:5.1%
Hung, *et al*. 2000 [13]	Hong Kong	6203 patients with HF Male age: 72.4 Female age: 77.5 Male: 44%	Retrospective 11 hospitals 1997	Total incidence: 0.7 per 1000 Age> 85: 20 per 1000(women); 14 per 1000 (men) Mortality:32%		
Sanderson, *et al*.1995 [14]	Hong Kong	730 patients with HF Male: 44%	Prospective	Incidence Women: 3.8/1000 Men: 3.0/1000	Hypertension:37% Ischemic heart disease:31% Valvular heart disease:15%	
Chong, *et al*. 2003 [15]	Malaysia	1435 acute medical admissions Male: 62.9% Mean age: 63.6 years	Retrospective Single-center	6.7%(97/1435)	Ischaemic heart disease: 49.5%(48) Hypertension: 18.6%(18) Dilated cardiomypathy: 4.1%(4) Valve disease: 4.1%(4) Anaemia:3.1%(3)	Aspirin: 55.7%(54) Warfarin:5.2%(5) Diuretics:96.9%(94) β-blocker:9.3%(9) Digoxin:13.4%(13) Nitrate:54.6%(53) ACEI: 43.3%(42) Statin:2.2%(2)
Ng, *et al*. 2003 [16]	Singapore	15774 admissions Age≥65 years Male: 46.6% Female: 53.4%	Retrospective 1991-1998	HF:4.5% HF 1991: 1436 HF 1997: 2457 Mortality:2.5%		
Koseki, *et al*. 2003 [17]	Japan	721 patients with HFPEF (EF≥50%) Age≥20years Male: 67.1%	Prospective multicenter	1-year mortality:8%		
Tsutsui, *et al*. 2007 [18]	Japan	2685 HF patients Age: 74(12) Male: 46%	Prospective multicenter 2004-2005	Mortality: 6.3% All-cause death: Hospital-HF:6.7% GP-HF:5.9% Cardiac death: Hospital-HF:2.9% GP-HF:1.7%	Aetiology: Ischemic: 30% Hypertension:35% Valvular:26% Cardiomyopathic:15% Comorbidities: AF: 40% Prior history of HF:83%	ACEI:31.5% ARB:30.9% ACEI or ARB:59.2% ACEI and ARB:3.3% β-blocker:27.4% Diuretics:62.0% Digitalis:43.0% CCB: 37.1%
Hamaguchi, *et al*. 2009 [20]	Japan 164 hospitals	2675 patients with HF Age: 71.5(13) Male: 58.1%	Prospective 2004-2007 Average follow-up:2.4 years		57% patients with HF had Anemia	
Tsuchihashi-Makaya, *et al*. 2009 [19]	Japan	1692 patients with HF Age ≥65: 69.3% Male: 59.7%	Prospective 2004-2007 Average follow-up: 2.4 years		Aetiology: Reduced EF: Ischemic:39.8% Hypertensive:21.6% Cardiomyopathic, dilated:36.3% Preserved EF: Ischemic:25.4% Hypertensive:44.3% Cardiomyopathic, dilated:5.1% (p<0.001) Comorbidities: Reduced EF: Hypertension:50.4% Hyperlipudemia:28.8% Atrial fibrillation:24.5% Preserved EF: Hypertension:68.3% Hyperlipudemia:22.8% Atrial fibrillation:38.3% (p<0.005)	
Shiba, *et al*. 2008 [21]	Japan	1,278 HF patients Age: 68.3(13.4) Male: 66%	Prospective 2000-2005 Mean follow-up:3.5 years		Aetiology: Coronary artery disease:25.4% Valvular heart disease: 26.4% Non-ischemic cardiomyopathy:28.6% Comorbidities: Hypertension:47.4% Diabetes:18.9% Dyslipidemia:16.7% AF:41.8%	
Shiba, *et al*. 2008 [21]	Japan	1,278 HF patients Age: 68.3(13.4) Male: 66%	Prospective 2000-2005 Mean follow-up:3.5 years		Aetiology: Coronary artery disease:25.4% Valvular heart disease: 26.4% Non-ischemic cardiomyopathy:28.6% Comorbidities: Hypertension:47.4% Diabetes:18.9% Dyslipidemia:16.7% AF:41.8%	
Shiba, *et al*. 2011 [22]	Japan	10,219 HF patients Age: 68.2(12.3) Male: 69.8%	Prospective Multicenter 2006-2010		Aetiology: Coronary artery disease: 47.1% Comorbidities: Hypertension:74.3% diabetes:23.3%	RAS inhibitors: 72.3% Diuretics :50.9% β-blockers:49.0% Digitalis23.5%
Hamaguchi, *et al*. 2011 [23]	Japan	2,675 patients with AF Age: 71(13) Male: 59.7%	Prospective 2004-2005 Average follow-up:2.1 years	NYHA class I:35.2%, II:55.2%, III:6.0%, IV: 3.6% All-cause death 14.7%(247) Cardiac death 8.8%(148) Rehospitalization 33.1%(557)	Aetiology: Ischemic:32% Valvular:27.7% Hypertensive:24.6% Dilated cardiomyopathy:18.2% Comorbidities: Hypertension：52.9% Hyperuricemia:46.8% Diabetes mellitus：29.9% Dyslipidemia:24.8% Prior stroke:15.0% COPD:6.7% Smoking:37.7% Prior MI:26.9% AF:35.2%	Aspirin: 47.2% Warfarin:40.8% Diuretics:88.2% β-blocker:48.6% Digitalis:30.9% Nitrate:23.3% ACEI: 37.4% ARB:44.4% ACEI/ARB:76.5% CCB:25.2% Statin:19.9%

GP: general practice; COPD: chronic obstructive pulmonary disease; CHF: chronic heart failure; HFPEF: heart failure with preserved ejection fraction; CCB: calcium channel blocker; ACEI: angiotensin-converting enzyme inhibitor; ARB: angiotensin-converting enzyme receptor inhibitor; RAS: renin-angiotensin system

**Table 1.** Prognosis of HF in East Asia

Author	Country	Patients (n)	Year	30-day Mortality	1-year Mortality	Mortality	Rehospitalization
Pei *et al*. [5]	China	7319	1993-2007	5.30%	*	*	*
Tseng *et al*. [11]	Taiwan	2692	2005	3.90%	*	*	*
Ng *et al*. [15]	Singapore	15774	1991-1998	*	*	2.50%	*
West *et al*. [39]	Singapore	1029(HFPEF)	2005-2009	*	*	2.20%	*
West *et al*. [39]	Thailand	989(HFPEF)	2005-2009	*	*	4.60%	*
Koseki *et al*. [16]	Japan	721(HFPEF)	2003	*	8%	*	23.4%
Tsutsui *et al*. [17]	Japan	2685	2004-2005	*	*	6.30%	40%
Hamaguchi *et al*. [19]	Japan	2675	2004-2005	*	*	8.8%	33.1%

HFPEF: Heart failure with preserved ejection fraction. *: Non-available data.
